# Lower resting-state EEG mean eigenvector centrality is associated with higher math anxiety

**DOI:** 10.3389/fnhum.2026.1843779

**Published:** 2026-06-02

**Authors:** Anna Pavlova, Timofey Adamovich, Sergey Malykh

**Affiliations:** 1 Cognitive Health and Intelligence Center, Institute of Cognitive Neuroscience, HSE University, Moscow, Russia; 2 FGBU Rossijskaa akademia obrazovania, Moscow, Russia

**Keywords:** eigenvector centrality, graph theory, healthy adolescents, math anxiety, resting state EEG

## Abstract

This study aimed to investigate resting-state neural correlates of math anxiety in high school students using a graph-theoretical approach. Sixty 10th-grade students (35 females; mean age = 16.15 years) participated in the study. Resting-state EEG data were acquired using a 32-channel wireless dry-sensor system (Cognionics Quick-32 series). Math anxiety was assessed using the Abbreviated Math Anxiety Scale. Mean eigenvector centrality was significantly and negatively associated with math anxiety in the alpha band during eyes-closed rest, the theta band during eyes-open rest, and the beta band during eyes-closed rest, with correlation coefficients ranging from *r* = −0.28 to −0.34. No significant associations were observed between centrality measures and sex. Overall, these findings are consistent with previous research linking reduced functional network integration to elevated math anxiety.

## 1 Introduction

Math anxiety (MA) is characterized by feelings of tension, fear, and apprehension when individuals engage with mathematical problems or numerical information (Moustafa et al., 2017). Numerous studies have documented a negative association between MA and mathematical achievement (see Zhang et al., 2019; Barroso et al., 2021, for meta-analyses). This relationship has been linked to factors such as negative math self-concept, unfavorable attitudes toward mathematics, and math avoidance (Ashcraft, 2002; Kaskens et al., 2020; Casanova et al., 2021).

Math anxiety has been linked to disruptions in executive functions, particularly working memory, as well as to difficulties in emotion regulation and inhibition of irrelevant information (Moustafa et al., 2017; Mammarella et al., 2017; Daches Cohen and Rubinsten, 2022). Although the neural mechanisms underlying MA are not yet fully understood, converging evidence suggests the involvement of multiple brain regions, including the prefrontal cortex, hippocampus, and amygdala, which support executive control, memory, and threat processing (Mah et al., 2016; Chin and Augustine, 2023). Together, these findings suggest that MA involves interactions between cognitive control and affective processing systems.

Mathematical cognition is supported by distributed neural systems, and disruptions in their interactions may contribute to the development of MA (Moustafa et al., 2017). In line with this view, Moustafa et al. (2019) proposed an integrated neural network framework for studying MA, highlighting the need to move beyond region-specific approaches. However, an established large-scale network model of MA is still lacking.

Graph-theoretical approaches provide a useful framework for examining MA-related differences in large-scale brain organization (Sporns, 2018; Beilock and Maloney, 2015). This perspective enables the quantification of global properties of network organization and the examination of how distributed brain systems interact to support both mathematical processing and its affective modulation. Graph-theoretical metrics of brain network topology (e.g., modularity, centrality, global efficiency) have been associated with anxiety-related traits and alterations in functional connectivity (Rodriguez, 2018; Yu et al., 2018; Tian et al., 2023), including in clinical populations such as social anxiety disorder (Zhang et al., 2022).

While there are a number of studies focusing on the neural correlates of MA during math task performance (see Artemenko et al., 2015 for a review), to our knowledge, only one study has investigated the relationship between MA and graph-theoretical measures of brain connectivity. Klados et al. (2017) assessed math anxiety using the Abbreviated Math Anxiety Scale in a large sample of students and recorded EEG activity in individuals with high (upper 15% of AMAS scores) and low (lower 15%) levels of MA. EEG was acquired prior to a series of arithmetic tasks, while participants were explicitly instructed that they would soon have to solve mathematical problems, thereby inducing anticipation of upcoming task performance during the recording. The authors reported that individuals with high math anxiety exhibited more efficient functional brain organization, which was interpreted as a compensatory mechanism related to emotion regulation.

Importantly, this design captures brain activity under anticipatory stress rather than in a true baseline condition. Thus, the observed network efficiency may reflect transient, context-dependent adaptations to expected task demands rather than stable properties of brain organization associated with MA. To date, no study has examined how baseline resting-state brain networks relate to MA. Unlike anticipation-based paradigms, resting-state networks capture baseline patterns that may predispose individuals to heightened emotional reactivity or altered cognitive control when confronted with mathematical demands. Therefore, examining graph-theoretical properties of resting-state networks may clarify whether MA is associated with enduring alterations in global network topology rather than solely context-dependent compensatory mechanisms.

1. This exploratory study aimed to address this gap by examining associations between resting-state brain network topology and math anxiety in adolescents. Specifically, we aimed to: test whether graph-theoretical metrics of large-scale brain networks are associated with individual differences in MA;
2. examine whether these associations depend on oscillatory frequency bands and resting-state conditions (eyes open vs. eyes closed);
3. assess whether graph-theoretical metrics associated with MA are related to sex, given robust evidence that girls report higher levels of MA than boys.

Understanding these intrinsic connectivity patterns may help identify stable neural markers underlying individual differences in MA and contribute to a more comprehensive neural model of math anxiety.

## 2 Materials and methods

### 2.1 Participants

Initially, 92 high school students participated in the study. Eligibility criteria included the absence of cognitive, neurological, or psychiatric diagnoses and brain injuries, as well as right-handedness. Due to artifacts in the EEG recordings and missing questionnaire data, the data from 32 participants were excluded from the analysis. The final sample consisted of 60 students (25 males, 35 females) 16–17 years old (*M* = 16.15, SD = 0.43, Median = 16).

The study consisted of two sessions. During the first session, participants completed in self-report socio-demographic questionnaire and Abbreviated Math Anxiety Scale questionnaire. During the second session, resting state EEG was recorded.

The study was approved by the Ethics Committee of the Psychological Institute of the Russian Academy of Education (protocol code 2020/4-1, date of approval 2 April 2020) in accordance with the Helsinki Declaration 2013. Written informed consent was obtained from all participants and their parents.

### 2.2 Instruments

Math anxiety was measured using an adapted version of the Abbreviated Math Anxiety Scale (AMAS) Hopko et al. (2003). The AMAS consists of 9 items, with 5 items comprising the learning math anxiety subscale (LMA) and 4 items comprising the math evaluation anxiety subscale (MEA). Each item presents a math-related situation, and the respondent rated the intensity of anxiety in that scenario on a 5-point Likert scale ranging from 1 (low anxiety) to 5 (high anxiety). In our previous work, the AMAS showed strong psychometric properties in Russian high school students (Marakshina et al., 2023). Demographic variables (age, sex and grade) were measured by self-report questionnaire. Math anxiety and demographic variables were measured during a class-based computer session.

### 2.3 EEG data acquisition and preprocessing

Resting-state EEG data were acquired over a 10-min session using a 32-channel wireless dry-sensor EEG headset (Cognionics Quick-32r) configured in a standard 10–20 montage and having a fixed sampling rate of 500 Hz per channel. A portable EEG system was used because data collection took place in a school setting. The Cognionics Quick-series systems employ active dry electrodes and high-input-impedance amplifiers designed to operate under elevated and variable electrode–skin impedance conditions, which are typical for dry EEG recordings. In contrast to conventional gel-based EEG, where impedance thresholds (e.g., <5–10 kΩ) are critical for signal quality, dry electrode systems rely on amplifier design and active shielding to maintain signal fidelity under substantially higher impedance levels.

Impedance quality was continuously monitored during acquisition using the system’s built-in real-time impedance tracking, which provides categorical feedback on electrode–scalp contact quality (e.g., “good” or “green” impedance status) rather than absolute impedance values. This approach is standard for wearable dry EEG systems and is intended to ensure stable recording conditions throughout data acquisition rather than to enforce wet-EEG impedance thresholds. Previous validation studies have demonstrated that Cognionics dry EEG systems yield EEG signals and event-related potentials highly comparable to conventional gel-based systems, with strong correspondence in spectral features and ERP morphology (*r* > 0.6–0.9), supporting their suitability for cognitive and developmental research in naturalistic environments (Hinrichs et al., 2020; Estepp et al., 2009).

Resting-state EEG was recorded in a quiet, empty classroom with the door closed. First, the participant’s skin was cleaned with ethanol to remove sebum from the skin surface and ensure high conductivity for electrodes. Electrode placement was iteratively adjusted until all channels achieved adequate contact quality, as indicated by the manufacturer’s green low-impedance status. Participants were instructed to maintain a relaxed state, minimize movement, and avoid falling asleep. The session consisted of five alternating two-minute blocks of eyes-open and eyes-closed conditions, cued by verbal instructions (Now open your eyes,” “Now close your eyes”) starting with eyes-closed condition. Participants could withdraw from the experiment at any time.

Preprocessing was performed using the MNE-Python and autoreject software packages. Initial preparation included identifying and removing non-physiological channels, interpolating bad channels, and re-referencing to the Common Average Reference (CAR). Data were then band-pass filtered (1–40 Hz), divided by condition (eyes open/closed), and segmented into one-second epochs with linear detrending. Artifact rejection began with a coarse epoch rejection based on a peak-to-peak amplitude threshold (> 125 muV). Subsequently, the data-driven sensor-repair algorithm, RANSAC, was applied to the remaining epochs to detect and correct problematic sensors. The final step involved applying the automated epoch-cleaning algorithm, AutoReject, for definitive data cleaning. Only the epochs surviving the AutoReject algorithm were retained in the analysis. Data cleaning resulted in the removal of approximately 40% of epochs and the interpolation of 3 channels per recording.

Functional connectivity estimation was conducted on the cleaned EEG epochs using the frites package, with data handling performed via MNE. The core metric employed was Gaussian-copula mutual information (GCMI), calculated to assess the non-linear functional coupling between all sensor pairs. GCMI has recently gained traction in neuroimaging analyses (e.g., Ince et al., 2017), including applications to EEG time-series (e.g., Thuraisingham, 2018). In contrast to commonly used connectivity metrics such as coherence, phase-locking value (PLV), or weighted phase lag index (wPLI), which predominantly capture linear relationships or phase-consistent coupling, GCMI quantifies statistical dependence in a more general sense and is therefore sensitive to both linear and non-linear interactions. This property is particularly relevant for resting-state EEG, where inter-regional interactions may not exhibit stable phase locking or be tied to external events. An additional advantage of GCMI lies in its copula-based formulation, which makes the measure invariant to marginal distributions. This is especially beneficial in the context of EEG, where signal amplitudes often deviate from Gaussianity, as it enhances robustness to distributional irregularities. For more details on GCMI in the context of resting state EEG data see our previous work (Pavlova et al., 2026).

This analysis was performed iteratively across four distinct frequency bands: Theta (4–8 Hz), Alpha (8–13 Hz), Low Beta (13–20 Hz), and High Beta (20–30 Hz). For each band, epochs were first band-pass filtered to isolate the specific range. The connectivity metric was calculated across all channels for every clean epoch, and the resulting connectivity matrices were subsequently averaged across all available epochs to yield a single, time-aggregated functional connectivity estimate per subject and per frequency band.

After computing the epoch-wise functional connectivity matrices for each subject and frequency band using Gaussian-copula mutual information, each matrix was thresholded at quantile levels of 0.5 and 0.8 per condition to create sparse weighted undirected graphs and all values below this threshold were removed. The choice of 0.5 and 0.8 quantile thresholds was motivated by the goal of examining network organization across different sparsity regimes, rather than relying on a single, potentially arbitrary cutoff. Specifically, the 0.5 threshold represents a relatively inclusive regime (retaining 50% of the strongest connections), consistent with our previous work (Zakharov et al., 2021), while the 0.8 threshold reflects a more stringent regime that isolates only the strongest functional connections (top 20%). This approach allows us to assess whether observed effects are stable across varying levels of network density. Importantly, we employed quantile-based thresholding rather than absolute cutoffs to account for inter-individual variability in overall connectivity strength.

Using the igraph Python library, the following graph metrics were computed at each threshold:

Characteristic path length was defined as the median shortest-path distance across all connected node pairs.

The clustering coefficient indexed the extent of local interconnectedness among neighboring nodes. Mean clustering coefficient was used in the analysis.

Eigenvector centrality indexed the extent to which a node was connected to other highly central nodes. Mean eigenvector centrality across all nodes was used in the study.

Betweenness centrality quantified a node’s influence in a network by measuring how often it lies on the shortest paths between pairs of other nodes, reflecting its potential to control or monitor information flow. Mean betweenness centrality was used in the analysis.

Participation coefficient quantified the diversity of a node’s intermodular connections and was computed using communities identified by the Leiden algorithm. Mean participation coefficient was used in the analysis.

Modularity described how well the network can be subdivided into non-overlapping groups of nodes or modules. Was computed via the Leiden algorithm.

The mean rich-club coefficient described the extent to which highly connected nodes formed a densely interconnected core.

### 2.4 Statistical analysis

Statistical analyses were conducted in Python (v3.14.0). Between-group differences were assessed using the Mann–Whitney *U* test. To examine associations between AMAS total score and graph-theoretical measures, Spearman rank-order correlations were computed with bootstrap resampling (*n* = 1,000) to derive 95% confidence intervals. In addition, a series of robust linear regression models were fitted using the RLM implementation from the statsmodels Python library. Each model included the AMAS total score as the dependent variable, a single graph-theoretical metric as the predictor, sex as a covariate, and their interaction (metric × sex). This approach allowed us to test whether the association between graph measures and mathematics anxiety differed by sex. Bootstrap resampling (*n* = 1,000) was applied to obtain confidence intervals for regression coefficients. For both correlation and regression analyses, effects were considered statistically significant when the 95% bootstrap confidence interval did not include zero.

This analytical strategy combined non-parametric testing, robust linear modeling, and bootstrap resampling to accommodate the non-normal and potentially heavy-tailed distributions typical of EEG-derived graph metrics. Robust regression was used to obtain stable parameter estimates in the presence of outliers and violations of model assumptions, while bootstrap confidence intervals provided a distribution-free and less assumption-dependent framework for statistical inference compared to conventional parametric tests or overly conservative multiple comparison corrections, such as FDR (see Westfall, 2011; Jeffries, 2007; Keselman et al., 2002).

## 3 Results

Given the significant associations with AMAS score were found for mean eigenvector centrality only, the results section focuses on that particular metric across frequency ranges (alpha, theta, beta low, beta high), eyes-open vs. eyes-closed conditions and 50% threshold vs. 80% threshold. Other graph metrics did not show consistent associations and are therefore not discussed in detail in the main text.

Table 1 presents descriptive statistics for math anxiety measures and mean eigenvector centrality measures. The AMAS total score showed a left-skewed distribution with a mean value of 16.57 (SD = 6.31), indicating generally low levels of math anxiety. The LMA subscale was similarly left-skewed with a mean value of 6.99 (SD = 3.41), whereas the MEA subscale approximated a normal distribution with a mean value of 9.59 (SD = 4.03). Mean eigenvector centrality values were roughly normally distributed, with slight right-skewness and occasional low-value outliers. Mean values for mean eigenvector centrality ranged from 0.32 to 0.48 (with SD range from 0.03 to 0.06) across frequency bands, open vs. closed eyes conditions and thresholds.

**Table 1:** Descriptive statistics of the math anxiety and mean eigenvector centrality measures.

Variable			Mean (SD)	Median	Min	Max	Skewness	Kurtosis
Math anxiety measures		AMAS total score	16.57 (6.31)	15.0	9	36	0.79	0.003
		AMAS LMA subscale	6.99 (3.41)	5.5	5	20	2.09	3.49
		AMAS MEA subscale	9.59 (4.03)	10.0	4	20	0.26	−0.55
Mean eigenvector centrality, alpha band	Eyes-closed	Threshold 0.5	0.47 (0.04)	0.47	0.36	0.54	−0.34	−0.34
		Threshold 0.8	0.34 (0.03)	0.35	0.27	0.42	−0.13	−0.76
	Eyes-open	Threshold 0.5	0.45 (0.04)	0.46	0.33	0.54	−0.61	0.37
		Threshold 0.8	0.32 (0.03)	0.33	0.25	0.40	−0.34	−0.44
Mean eigenvector centrality, theta band	Eyes-closed	Threshold 0.5	0.47 (0.05)	0.46	0.32	0.58	−0.28	0.34
		Threshold 0.8	0.33 (0.04)	0.33	0.24	0.42	−0.18	−0.38
	Eyes-open	Threshold 0.5	0.45 (0.05)	0.46	0.32	0.55	−0.64	0.48
		Threshold 0.8	0.32 (0.04)	0.33	0.23	0.40	−0.29	−0.10
Mean eigenvector centrality, low beta band	Eyes-closed	Threshold 0.5	0.48 (0.04)	0.48	0.38	0.56	−0.26	−0.27
		Threshold 0.8	0.35 (0.04)	0.35	0.27	0.41	−0.11	−0.88
	Eyes-open	Threshold 0.5	0.47 (0.06)	0.47	0.33	0.71	0.81	3.90
		Threshold 0.8	0.33 (0.04)	0.33	0.23	0.47	0.42	0.87
Mean eigenvector centrality, low beta band	Eyes-closed	Threshold 0.5	0.44 (0.05)	0.45	0.32	0.56	−0.25	0.35
		Threshold 0.8	0.32 (0.03)	0.32	0.25	0.40	−0.03	0.13
	Eyes-open	Threshold 0.5	0.41 (0.06)	0.42	0.27	0.53	−0.35	−0.43
		Threshold 0.8	0.30 (0.05)	0.30	0.17	0.40	−0.42	−0.08

*N* = 60.

Table 2 provides information on sex differences for all the variables included in the final analysis. Overall, no sex differences were observed for both math anxiety and mean eigenvector centrality measures.

**Table 2:** Sex differences in the behavioral and mean eigenvector centrality measures.

Variable			Mean (SD) Male	Mean (SD) Female	Mean difference	Mann–Whitney stats	*p*-value
Math anxiety measures		AMAS total score	16.53 (6.04)	16.83 (6.53)	−0.68	509.5	0.69
		AMAS LMA subscale	7.19 (3.57)	6.86 (3.35)	0.34	537.5	0.91
		AMAS MEA subscale	8.96 (3.22)	9.97 (4.45)	−1.01	481.0	0.41
Mean eigenvector centrality, alpha band	Eyes-closed	Threshold 0.5	0.47 (0.03)	0.47 (0.04)	0.00	418.0	0.78
		Threshold 0.8	0.34 (0.03)	0.33 (0.03)	0.01	506.0	0.31
	Eyes-open	Threshold 0.5	0.46 (0.04)	0.45 (0.05)	0.01	596.0	0.94
		Threshold 0.8	0.32 (0.03)	0.32 (0.03)	0.00	482.0	0.62
Mean eigenvector centrality, theta band	Eyes-closed	Threshold 0.5	0.46 (0.04)	0.47 (0.05)	−0.01	431.0	0.25
		Threshold 0.8	0.33 (0.03)	0.33 (0.04)	0.00	453.0	0.38
	Eyes-open	Threshold 0.5	0.44 (0.05)	0.46 (0.06)	−0.01	443.0	0.20
		Threshold 0.8	0.32 (0.04)	0.32 (0.03)	0.00	396.0	0.54
Mean eigenvector centrality, low beta band	Eyes-closed	Threshold 0.5	0.49 (0.03)	0.48 (0.04)	0.01	507.0	0.30
		Threshold 0.8	0.36 (0.03)	0.35 (0.03)	0.01	532.0	0.16
	Eyes-open	Threshold 0.5	0.48 (0.04)	0.47 (0.06)	0.01	598.0	0.31
		Threshold 0.8	0.34 (0.04)	0.33 (0.04)	0.01	582.0	0.42
Mean eigenvector centrality, high beta band	Eyes-closed	Threshold 0.5	0.44 (0.04)	0.44 (0.05)	0.00	498.0	0.70
		Threshold 0.8	0.32 (0.03)	0.32 (0.04)	0.00	494.0	0.74
	Eyes-open	Threshold 0.5	0.42 (0.06)	0.41 (0.06)	0.01	554.0	0.56
		Threshold 0.8	0.30 (0.04)	0.30 (0.05)	0.00	557.0	0.61

*N* = 60.

Table 3 presents the Spearman correlation coefficients between total AMAS score and mean eigenvector centrality measures. Overall, a negative association between mean eigenvector centrality and total AMAS score were observed at both the 50 and 80% thresholds for alpha (only eyes-closed condition) and theta (only eyes-open condition) frequency ranges, while for low-beta band (both eyes-open and eyes-closed conditions) such an association was observed for 50%, but not for 80% threshold. The observed associations were small to moderate in magnitude (r ranged from −0.28 to −0.34).

**Table 3:** Spearman correlations between mean eigenvector centrality measures and AMAS total score.

Mean eigenvector centrality measure	Threshold 50%			Threshold 80%		
	Spearman r	*p*-value	Bootstrap 95% CI	Spearman r	*p*-value	Bootstrap 95% CI
Alpha band, eyes closed	−0.34	0.009**	[−0.57, −0.13]	−0.28	0.03*	[−0.53, −0.04]
Alpha band, eyes open	−0.16	0.22	[−0.41, 0.10]	−0.11	0.41	[−0.34, 0.15]
Theta band, eyes closed	−0.20	0.13	[−0.44, 0.04]	0.15	0.25	[−0.40, 0.10]
Theta band, eyes open	−0.31	0.02*	[−0.53, −0.07]	−0.32	0.01*	[−0.55, −0.09]
Low beta band, eyes closed	−0.29	0.03*	[−0.54, −0.01]	−0.23	0.08	[−0.50, 0.04]
Low beta band, eyes open	−0.28	0.03*	[−0.50, −0.04]	−0.22	0.07	[−0.46, 0.01]
High beta band, eyes closed	−0.11	0.38	[−0.39, 0.16]	−0.13	0.33	[−0.40, 0.14]
High beta band, eyes open	0.10	0.47	[−0.14, 0.33]	0.03	0.79	[−0.20, 0.27]

*N* = 60; *p-value < 0.05; **p-value < 0.01.

Table 4 demonstrates the results of robust linear regressions with an interaction between mean eigenvector centrality and sex. While the standardized robust linear regression beta coefficients for mean eigenvector centrality demonstrate the effects similar in direction and magnitude to the Spearman correlations, neither sex nor interaction between sex and mean eigenvector centrality was statistically significant, suggesting the absence of gender-specific effects.

**Table 4:** Bootstrapped robust linear regression for AMAS and mean eigenvector centrality measures including the interaction with sex.

Mean eigenvector centrality type, included in the model	Variables	Threshold 50%			Threshold 80%		
		Standardized beta	Bootstrap 95% CI	*p*-val	Standardized beta	Bootstrap 95% CI	*p*-val
Alpha band, eyes closed	Mean eigenvector centrality	−0.31	[−0.68, −0.07]	0.02*	−0.30	[−0.65, −0.05]	0.04*
	Sex	0.03	[−0.22, 0.29]	0.84	−0.02	[−0.28, 0.25]	0.87
	Interaction	0.00	[−0.30, 0.32]	0.98	0.05	[−0.28, 0.33]	0.75
Alpha band, eyes open	Mean eigenvector centrality	−0.09	[−0.38, 0.20]	0.50	−0.07	[−0.34, 0.20]	0.61
	Sex	0.01	[−0.24, 0.28]	0.92	0.02	[−0.27, 0.28]	0.90
	Interaction	−0.08	[−0.38, 0.29]	0.95	0.01	[−0.34, 0.27]	0.92
Theta band, eyes closed	Mean eigenvector centrality	−0.16	[−0.50, 0.03]	0.21	−0.14	[−0.43, 0.10]	0.30
	Sex	0.03	[−0.22, 0.28]	0.76	0.03	[−0.24, 0.27]	0.80
	Interaction	−0.04	[−0.28, 0.27]	0.76	−0.03	[−0.26, 0.28]	0.80
Theta band, eyes open	Mean eigenvector centrality	−0.23	[−0.65, −0.03]	0.04*	−0.26	[−0.69, −0.06]	0.04*
	Sex	0.04	[−0.23, 0.30]	0.77	0.03	[−0.24, 0.27]	0.80
	Interaction	−0.03	[−0.23, 0.30]	0.83	−0.02	[−0.36, 0.25]	0.86
Low beta band, eyes closed	Mean eigenvector centrality	−0.30	[−0.61, 0.04]	0.03*	−0.24	[−0.57, 0.08]	0.07
	Sex	−0.02	[−0.30, 0.22]	0.87	−0.02	[−0.31, 0.22]	0.87
	Interaction	−0.04	[−0.42, 0.23]	0.76	−0.02	[−0.33, 0.30]	0.91
Low beta band, eyes open	Mean eigenvector centrality	−0.25	[−0.62, −0.01]	0.04*	−0.21	[−0.59, 0.06]	0.12
	Sex	−0.02	[−0.33, 0.22]	0.85	0.00	[−0.30, 0.23]	0.99
	Interaction	−0.01	[−0.32, 0.30]	0.95	−0.02	[−0.36, 0.26]	0.87
High beta band, eyes closed	Mean eigenvector centrality	0.02	[−0.44, 0.38]	0.86	−0.01	[−0.48, 0.33]	0.99
	Sex	0.00	[−0.23, 0.28]	0.99	0.01	[−0.23, 0.27]	0.95
	Interaction	−0.22	[−0.67, 0.28]	0.11	−0.13	[−0.55, 0.33]	0.38
High beta band, eyes open	Mean eigenvector centrality	0.14	[−0.15, 0.42]	0.27	0.06	[−0.22, 0.32]	0.68
	Sex	0.01	[−0.24, 0.28]	0.97	0.02	[−0.24, 0.29]	0.88
	Interaction	−0.15	[−0.44, 0.20]	0.25	0.05	[−0.35, 0.26]	0.75

*N* = 60; *p-value < 0.05.

## 4 Discussion

To our knowledge, the present study is the first to examine resting-state whole-brain network properties associated with math anxiety using a graph-theoretical approach.

### 4.1 Association between EEG measures and math anxiety

We found that lower mean eigenvector centrality was associated with higher self-reported math anxiety, with effects in the small-to-moderate range. These associations were frequency- and state-specific, emerging in the alpha band during eyes-closed rest, the theta band during eyes-open rest, and the low beta band during both eyes-closed and eyes-open rest.

Notably, among the examined graph-theoretical metrics, mean eigenvector centrality was the only measure that showed consistent associations with math anxiety. This pattern may reflect differences in the aspects of network organization captured by distinct graph measures, rather than the exclusive relevance of a single metric. Eigenvector centrality is a graph-theoretical measure reflecting the extent to which a node is connected to other highly central nodes within the network, thereby capturing its global embedding and influence (Lohmann et al., 2010; van den Heuvel and Sporns, 2011). Although eigenvector centrality is not a direct measure of integration in the same sense as global efficiency or characteristic path length, it is often considered a complementary index of hub connectivity (Sporns, 2018). In the present study, we focused on mean eigenvector centrality, computed by averaging node-level values to obtain a network-level summary. When aggregated across nodes, this measure may be interpreted as a coarse index of whole-brain network organization within the functional connectome (Zuo et al., 2012). Higher mean eigenvector centrality may therefore reflect greater global integration of influential network nodes. Taken together, the present findings suggest that lower large-scale network integration at rest is associated with higher levels of math anxiety.

Interestingly, this pattern contrasts with the findings reported by Klados et al. (2017), who observed higher network efficiency in individuals with elevated math anxiety. Presumably, the difference in study designs accounts for this discrepancy. While Klados et al. (2017) recorded brain activity under explicit anticipation of upcoming math tasks, the present study examined intrinsic resting-state activity in the absence of task-related demands. One possible interpretation is that increased network efficiency in highly math-anxious individuals reflects a context-dependent, compensatory reconfiguration of brain networks in response to anticipated stress, potentially supporting heightened vigilance or emotion regulation. In contrast, reduced mean eigenvector centrality at rest, as observed in the present study, may indicate lower baseline integration of large-scale brain networks. Given that resting-state brain activity has been shown to exhibit moderate-to-high test–retest reliability across sessions and over extended time intervals (Rolle et al., 2022; Isaza et al., 2026) we interpret it as a more stable, trait-like feature. However, cross-sectional design of the study does not allow to formally disentangle state-dependent and trait-like contributions, and this interpretation should therefore be considered tentative. Longitudinal studies are needed to directly test and validate the extent to which resting-state EEG markers reflect stable individual traits versus transient state fluctuations over time.

The observed association between eigenvector centrality and math anxiety can be interpreted within the framework of two prominent theoretical models of math anxiety: the Deficit (Reduced Competence) Theory and the Debilitating Anxiety (Processing Efficiency) Model (Carey et al., 2016). Although these models differ in their proposed primary mechanisms, both assume that math anxiety emerges from inefficiencies in cognitive–affective processing that place increased demands on distributed neural systems. From a network neuroscience perspective, such inefficiencies are expected to manifest as alterations in large-scale network integration, which supports both cognitive resource allocation and emotion regulation.

The Deficit Theory proposes that math anxiety emerges as a consequence of an initial deficit in cognitive resources (Ramirez et al., 2018). Consistent with this view, a growing body of evidence highlights the importance of efficient large-scale information flow for cognitive functioning. In particular, global network integration has been associated with higher working memory capacity (Cohen and D’Esposito, 2016), inhibitory control (Marek et al., 2015), executive functioning (Breedt et al., 2023), cognitive reserve (Pappalettera et al., 2024), and intelligence (Langer et al., 2012). From this perspective, reduced eigenvector centrality may be consistent with less efficient large-scale network integration supporting cognitive control that predispose individuals to the development of math anxiety.

In contrast, the Debilitating Anxiety or Processing Efficiency Model emphasizes non-cognitive origins of math anxiety, such as difficulties in emotion regulation, which interfere with cognitive performance (Eysenck and Calvo, 1992). Importantly, large-scale network integration has also been implicated in emotion regulation processes (see Pessoa, 2017 for a review). For example, whole-brain network integration increases during active emotion regulation (Brandl et al., 2018), while greater network efficiency at rest has been associated with the use of expressive suppression—an emotion regulation strategy aimed at modifying emotional responses (Pan et al., 2018). Accordingly, the negative association between eigenvector centrality and math anxiety observed in the present study may also be consistent with less efficient large-scale network organization supporting emotion regulation in math-related contexts.

Importantly, cognitive abilities and emotion regulation capacities are not independent but strongly interrelated processes. Executive functions such as working memory and inhibitory control are critically involved in successful emotion regulation, while emotion regulation demands, in turn, place additional load on domain-general cognitive resources (Ochsner and Gross, 2005; Diamond, 2013). At the neural level, both cognitive control and emotion regulation rely on coordinated interactions within distributed brain networks (Pessoa, 2017). From this perspective, large-scale network integration may represent a shared neural infrastructure supporting the dynamic interplay between cognitive and affective processes. Accordingly, reduced eigenvector centrality could reflect less efficient integration of systems jointly contributing to cognitive performance and emotion regulation, thereby increasing vulnerability to math anxiety. This interpretation remains speculative, as the present data do not allow direct assessment of these processes, but it provides a unifying framework linking deficit- and anxiety-based accounts of math anxiety in accordance with a theory of bidirectional association between math achievements and math anxiety (Carey et al., 2016). Future studies combining graph-theoretical network metrics with comprehensive cognitive and affective assessments, as well as longitudinal designs, will be essential to clarify the relative contribution of these mechanisms.

### 4.2 The frequency- and state-specific nature of the association

Notably, the frequency- and state-specific nature of the observed effects suggests that math anxiety is associated with alterations in neural network organization under specific modes of cognitive readiness rather than reflecting a global, state-independent property of brain networks. Alpha-band networks during eyes-closed rest have been shown to dominate resting-state topology and are thought to support internally oriented processing and the coordination of widespread cortical regions (Klimesch, 1999; Micheloyannis et al., 2006; Lou et al., 2025). Reduced eigenvector centrality within alpha-band networks may therefore indicate less efficient integration of influential nodes during internally driven cognitive and affective states, which are particularly relevant for anticipatory and ruminative components of math anxiety.

Theta-band network topology has been consistently linked to cognitive control, conflict monitoring, trait anxiety and affective–cognitive interactions (Zou et al., 2012; Badre and Nee, 2018; Pscherer et al., 2020; Iyer et al., 2020; Imperatori et al., 2019). The observed association between math anxiety and theta-band eigenvector centrality during eyes-open rest may reflect altered coordination of control-related hubs under conditions of heightened external engagement and ongoing internal monitoring.

Finally, beta-band networks have been associated with externally oriented readiness, maintenance of cognitive set, and vigilance. Previous graph-based EEG studies indicate that beta-band topology contributes to large-scale network integration (Micheloyannis et al., 2006; Stam, 2014). In this context, reduced eigenvector centrality in the beta band during both eyes-closed and eyes-open rest may reflect altered large-scale coordination supporting sustained cognitive preparedness.

### 4.3 The link between mean eigenvector centrality and sex

Notably, no sex differences in AMAS scores or connectivity measures were observed in the present sample, despite previous reports of higher math anxiety in females (Devine et al., 2012; Van Mier et al., 2019). One possible explanation for this discrepancy may lie in the specific characteristics of our sample, which consisted of students enrolled in university preparatory courses. This group likely represents a more academically motivated population, in which typical sex differences may be attenuated due to self-selection processes and more homogeneous levels of academic engagement. However, this interpretation remains tentative, and alternative explanations, such as limited statistical power to detect small effects, cannot be ruled out.

Moreover, the association between mean eigenvector centrality and math anxiety did not differ between males and females. This finding is consistent with a growing body of literature suggesting that large-scale brain network topology is largely comparable between males and females, particularly with respect to global graph-theoretical properties (Joel et al., 2015; Eliot et al., 2021). Moreover, previous research indicates that sex differences in anxiety and related cognitive–affective traits are more strongly shaped by sociocultural influences and learning experiences than by intrinsic differences in large-scale brain organization (Devine et al., 2012; Van Mier et al., 2019).

At the same time, emerging neuroimaging evidence suggests that sex differences in the neural correlates of math anxiety may manifest at more fine-grained or region-specific levels rather than in global network measures. For instance, Gonzalez et al. (2019) demonstrated sex-specific patterns of neural activation associated with math anxiety during task performance, implicating partially distinct cortical regions in males and females. Together, these findings suggest that while sex-related differences in math anxiety may be reflected in localized or task-evoked neural dynamics, the global network-level architecture indexed by mean eigenvector centrality may capture a largely sex-independent mechanism underlying trait-like anxiety.

Several limitations of the present study should be acknowledged. First, the cross-sectional design precludes causal inferences regarding the relationship between large-scale network topology and math anxiety. Second, some associations were modest in size and would likely not survive stringent correction for multiple comparisons, warranting caution in their interpretation and highlighting the need for replication in larger samples. Third, some results showed sensitivity to the choice of network threshold. Specifically, in the low beta frequency band, the observed association was statistically significant at the more inclusive threshold (50%) but not at the more stringent threshold (80%). While such differences do not necessarily indicate a lack of robustness—given that thresholding can differentially retain moderate versus only the strongest connections—they underscore the importance of cautious interpretation and the need to consider threshold-dependent effects in future work.

In conclusion, the present study provides evidence that that math anxiety is associated with frequency- and state-specific alterations in large-scale resting-state brain network topology, as indexed by reduced eigenvector centrality, independent of sex. By focusing on global network organization, this work extends existing research on the neural correlates of math anxiety and situates the phenomenon within a network neuroscience framework. These findings underscore the importance of considering frequency-specific brain network topology in future investigations of math anxiety and related affective–cognitive traits.

## Data Availability

The raw data supporting the conclusions of this article will be made available by the authors, without undue reservation.
